# Detection of BCR-ABL Positive Cells in an Asymptomatic Patient: A Case Report and Literature Review

**DOI:** 10.1155/2010/939706

**Published:** 2010-02-18

**Authors:** Soley Bayraktar, Mark Goodman

**Affiliations:** Slyvester Comprehensive Cancer Center, 1475 NW Ave, Suite 3300, Miami, FL 33136, USA

## Abstract

We report a case of an asymptomatic 39-year-old male who was incidentally found to have a white blood cell count of 15 000/mm^3^ associated with a positive BCR-ABL/*t*(9;22)(q34;q11) chromosomal translocation detected in 51/300 of cells by FISH and RT-PCR from peripheral blood. Within the next 3 months, leukocytosis spontaneously subsided; however, BCR-ABL by RT-PCR and FISH was persistent both in peripheral blood and bone marrow. The patient was not started on any therapy and is being followed regularly with laboratory checkup and physical examination for monitoring signs and symptoms of chronic myeloid leukemia (CML) and biological behavior of his BCR-ABL transcripts. At 1 year of surveillance, he is disease free; however he has persistent detection of BCR-ABL fusion gene. Our case is challenging because actual risk of developing CML in BCR-ABL positive healthy, asymptomatic patients is not known.

## 1. Introduction

Chronic myeloid leukemia (CML) is a myeloproliferative disorder with an incidence of 2 cases per 100,000 people and represents 7–20% of all leukemia cases [1]. Although is an uncommon entity, CML is one of the few leukemias that has a defined pathogenesis; which involves the Philadelphia chromosome (Ph)/*t*(9;22)(q34;q11) chromosomal translocation and activated tyrosine kinases (TK) in the mutated hematopoietic stem cell [2]. Also, it is one of the first leukemias in which a rational targeted therapy with tyrosine kinase inhibitors (TKI) revolutionized the treatment and natural history of the disease by improving overall survival (OS) with long term remissions [1, 3]. Although it was once to be thought to be pathognomonic for CML, evidence of BCR-ABL fusion gene or *t*(9;22)(q34;q11) translocation is also found in approximately in one-third of cases of ALL and <1% of AMLs [4, 5]. Moreover, there is a substantial group of asymptomatic, healthy people who can bare this chromosomal aberration and never develop CML [5–7]. As of now, there are no guidelines for stratification of the risk factors for CML development, monitoring BCR-ABL transcripts and establishing a proper treatment in this subgroup. We report a case of an asymptomatic 39-year-old male who was found to have white blood cell (WBC) count of 15.000/mm^3^ without therapy yet, associated with a positive BCR-ABL translocation and subsequent normalization of his leukocytosis with persistence of the BCR-ABL detection in peripheral blood (PB) and bone marrow (BM) cells. We provide a detailed clinical analysis in conjunction with a brief literature review.

## 2. Case Report

The patient is a 39-year-old Jamaican man who was found to have elevated WBC of 15.000/mm^3^ with normal differential, as an incidental finding during a followup visit to his primary care physician. Review of systems were negative including fever/chills, weight loss, night sweats and bleeding. Past medical history was significant for hypertension and diabetes mellitus type II. On physical examination, there were no palpable enlarged lymph nodes, organomegaly or signs of bleeding. Complete Blood Count (CBC) showed WBC of 15.000/mm^3^ with normal differential, hemoglobin (Hb) and hematocrit (Htc) of 15 mg/dL and 45%, and platelets of 308.000/mm^3^. The peripheral blood smear showed normal neutrophils, no myelocytes, metamyelocytes, nucleated red blood cells or giant platelets. Metaphase cytogenetic analysis of PB did not demonstrate any karyotype abnormalities; however BCR/ABL *t*(9;22) was detected in 51 of 300 cells by Fluorescence In-Situ Hybridization (FISH) and quantitative real time PCR (RT-PCR). There were no evidence of other cytogenetic or molecular abnormalities. Further work-up with CT-chest/abdomen/pelvis did not reveal any systemic disease. At that time, no specific treatment was recommended and a follow-up appointment was scheduled. 

During 3 months surveillance, he had been asymptomatic and physical examination was normal. A repeat CBC showed WBC of 7600/mm^3^ with 78% neutrophils, 16% lymphocytes, 5.9% basophils, 3.4% monocytes without metamyelocytes, myelocytes or any other atypical granulocytes. Hb and platelets were 15.3 mg/dL and 364.000/mm^3^, respectively. His metabolic panel was within normal limits, including LDH of 588 mg/Dl. Peripheral blood smear (PBS) and flow cytometry was unremarkable again. The repeat BCR/ABL *t*(9;22) FISH and PCR analysis was positive. Further work-up with bone marrow biopsy demonstrated normocellular BM with granulocytes in all maturation stages with adequate number and normal morphology; and 2% blasts. The M  :  E ratio was normal. Karyotype analysis of bone marrow showed *t*(9;22) in 4 of 20 metaphases. Furthermore, FISH for BCR/ABL *t*(9;22) translocation was positive in 52% of the bone marrow cells. Currently, at 1 year of surveillance, the patient still continues being asymptomatic without any treatment and there is no cytological progression concordant with this evident genetic aberration.

## 3. Discussion

CML is a rare myeloproliferative disorder with approximately 4000 new cases diagnosed yearly in USA [8]. More than 50% of patients with CML present during the chronic phase of the disease with florid constitutional symptoms and signs including hepatosplenomegaly, marked leukocytosis (usually >25.000/mm^3^) with or without thrombocytosis and basophilia, and a hypercellular bone marrow with prevalence of myeloid cell population [1]. On the other hand, up to 40% of patients with chronic phase CML are totally asymptomatic patients and are diagnosed incidentally by routine laboratory tests [8]. 

Although demonstration of the BCR-ABL fusion gene or chromosomal translocation *t*(9;22)(q34;q11) in PB and/or BM cells is part of the essential criteria for CML diagnosis [2, 5], there is a significant prevalence of positive extremely low levels of BCR-ABL transcripts (accounting for no more than 1–10 per 10^8^ WBC) up to 30% of completely asymptomatic healthy subjects with normal blood counts [5, 6]. This observation suggests that BCR-ABL has to be expressed in the appropriate progenitor cells in order to gain a selective growth advantage and cause clinical disease [9]. Furthermore, Biernaux et al. [6] has demonstrated increasing incidence of positive BCR-ABL transcripts by advancing age as transcripts were not detectable in cord blood and in only 1 of 22 children but in 18 of 52 adults. The effects of age could be explained by the fact that, in adults, there have been more cell divisions and therefore a greater chance of accumulating genetic lesions [6]. 

Our patient's presentation leads to a complex debate in terms of the clinical management and followup of his “condition”. Initially, he presented with mildly elevated WBC with no signs or symptoms of CML, but with a positive BCR-ABL; subsequently, his blood counts normalized without any treatment; he remained asymptomatic, but BCR-ABL in PB and BM persisted. 

FISH is an essential technique for the cytogenetic identification of the Ph chromosome; however, different groups have reported a FISH sensitivity of 98% with false positive results of 2.3 to 2.8% [10]. These low rates of false positive detections are usually clinically non-significant, when the FISH analysis is backed by cytogenetic metaphase analysis and RT-PCR of PB or BM cells. RT-PCR measures the BCR-ABL mRNA transcripts comparing them with a housekeeping gene that ideally is expressed in leukemia cells at exact same level as in normal cells [11]. The original descriptions of positive BCR-ABL detections in healthy persons were done using a exquisite sensible RT-PCR technique 40 times more sensible that the usual clinical test [5, 6]. In our clinical case, the patient had serial RT-PCR assessments done by different laboratories in a short time frame, both PB and BM were positive; so regardless of the sensitivity of the RT-PCR, the sequential unbiased followup decreases substantially the rate of false positive results in his samples. 

The most challenging question is the proper approach to the treatment and monitoring of the patient presented in our clinical case. CML treatment has radically changed with the introduction of TKI in 1998 [12]. TKI have demonstrated excellent responses with increase in OS and mild adverse effects [3, 12]. Also, it has been demonstrated that treatments in patients with CML, including TKI like imatinib, are more effective in the chronic phase of the disease than in advanced stages [9]. Finally but not least, it is very important to take into account that untreated CML is characterized by the inevitable transition from a chronic phase to an accelerated phase and on to blast crisis in a median time of 4 years [1]. On the other hand, since the incidence of asymptomatic healthy individuals that harbor these aberrations exceeds by far the incidence of CML, most positive subjects will never develop leukemia [5, 7] and, to our knowledge there are no guidelines or reports about the treatment of asymptomatic patients with positive BCR-ABL fusion gene, without any other features of CML; similar to the clinical situation of our patient. We are facing an important therapeutic dichotomy: should we treat the patient using his persistent positive BCR-ABL results as the unique component of disease, even though he has no other CML features, exposing him to unnecessary medication adverse effects, without a defined therapeutic goal or should we pursue a “watch and wait” approach as commonly used in chronic lymphocytic leukemia, implementing close followups including CBC, cytogenetics, FISH and RT-PCR to monitor the evolution of the BCR-ABL transcripts and the eventual development of a full blown leukemia? 

Since our patient is totally asymptomatic, with normal blood counts with low persistent detection of BCR-ABL fusion gene, the consensus was to follow him without treatment and very close control of his cell counts with special attention to his BCR-ABL fusion gene evolution every three months, as it is usually done for CML patients in treatment.

## 4. Conclusion

Asymptomatic healthy persons with *t*(9;22)(q34;q11) chromosomal translocation are fairly common. Although there is no long-term followup for this group of patients, given the rarity of CML in general population, one could extrapolate that only small portion of this population would develop CML. To our knowledge, there has been no report of an asymptomatic patient with persistent positive BCR-ABL translocation and initially elevated WBC that normalized without therapy. Currently, the biological consequences of positive BCR-ABL in healthy persons without histological evidence of leukemia have not been studied in detail and there is no standard approach for treatment.
